# Local transmission and global dissemination of New Delhi Metallo-Beta-Lactamase (NDM): a whole genome analysis

**DOI:** 10.1186/s12864-016-2740-0

**Published:** 2016-06-13

**Authors:** Wei Xin Khong, Eryu Xia, Kalisvar Marimuthu, Wenting Xu, Yik-Ying Teo, Eng Lee Tan, Shiyong Neo, Prabha Unny Krishnan, Brenda S. P. Ang, David C. B. Lye, Angela L. P. Chow, Rick Twee-Hee Ong, Oon Tek Ng

**Affiliations:** Institute of Infectious Disease and Epidemiology, Communicable Disease Centre, Tan Tock Seng Hospital, Singapore, Singapore; NUS Graduate School for Integrative Science and Engineering, National University of Singapore, Singapore, Singapore; Yong Loo Lin School of Medicine, National University of Singapore, Singapore, Singapore; Saw Swee Hock School of Public Health, National University of Singapore, Singapore, Singapore; Life Sciences Institute, National University of Singapore, Singapore, Singapore; Department of Statistics and Applied Probability, National University of Singapore, Singapore, Singapore; Genome Institute of Singapore, Agency for Science, Technology and Research, Singapore, Singapore; Centre for Biomedical and Life Sciences, Singapore Polytechnic, Singapore, Singapore; Department of Pediatrics, University Children’s Medical Institute, National University of Singapore, Singapore, Singapore; Department of Laboratory Medicine, Tan Tock Seng Hospital, Singapore, Singapore; Communicable Disease Centre, Tan Tock Seng Hospital, 11, Jalan Tan Tock Seng, Singapore, 308433 Singapore

## Abstract

**Background:**

New Delhi metallo-β-lactamase (*bla*_NDM_), a plasmid-borne carbapenemase gene associated with significant mortality and severely limited treatment options, is of global public health concern as it is found in extremely diverse Gram-negative bacterial strains. This study thus aims to genetically characterize local and global spread of *bla*_NDM_.

**Methods:**

To investigate local transmission patterns in the context of a single hospital, whole genome sequencing data of the first 11 *bla*_NDM_-positive bacteria isolated in a local hospital were analyzed to: (1) identify and compare *bla*_NDM_-positive plasmids; and (2) study the phylogenetic relationship of the bacteria chromosomes. The global analysis was conducted by analyzing 2749 complete plasmid sequences (including 39 *bla*_NDM_-positive plasmids) in the NCBI database, where: (1) the plasmids were clustered based on their gene composition similarity; (2) phylogenetic study was conducted for each *bla*_NDM_-positive plasmid cluster to infer the phylogenetic relationship within each cluster; (3) gene transposition events introducing *bla*_NDM_ into different plasmid backbones were identified; and (4) clustering pattern was correlated with the plasmids’ incompatibility group and geographical distribution.

**Results:**

Analysis of the first 11 *bla*_NDM_-positive isolates from a single hospital revealed very low *bla*_NDM_-positive plasmid diversity. Local transmission was characterized by clonal spread of a predominant plasmid with 2 sporadic instances of plasmid introduction. In contrast to the low diversity locally, global *bla*_NDM_ spread involved marked plasmid diversity with no predominant bacterial clone. Thirty-nine (1.4 %) out of the 2749 complete plasmid sequences were *bla*_NDM_-positive, and could be resolved into 7 clusters, which were associated with plasmid incompatibility group and geographical distribution. The *bla*_NDM_ gene module was witnessed to mobilize between different plasmid backbones on at least 6 independent occasions.

**Conclusions:**

Our analysis revealed the complex genetic pathways of *bla*_NDM_ spread, with global dissemination characterized mainly by transposition of the *bla*_NDM_ gene cassette into varied plasmids. Early local transmission following plasmid introduction is characterized by plasmid conjugation and bacterial spread. Our findings emphasize the importance of plasmid molecular epidemiology in understanding *bla*_NDM_ spread.

**Electronic supplementary material:**

The online version of this article (doi:10.1186/s12864-016-2740-0) contains supplementary material, which is available to authorized users.

## Background

The emergence of carbapenemase-producing *Enterobacteriaceae* (CPE) has become an important threat to global health. CPE are primarily recognized in health care settings [1], with the prevalence from clinical samples increasing globally [2–6]. Outcomes of CPE infections are poor, where mortality associated with infections can reach over 40 % [7, 8]. With the widespread dissemination of extended-spectrum β-lactamases, carbapenems are the last class of safe and effective antimicrobials for treating multidrug-resistant Gram-negative bacterial infections, the effectiveness of which has been greatly undermined by CPE [9]. As a result, there is a pressing need to understand the transmission pathways of carbapenemases to inform infection control, the main intervention available against CPE transmission and infection.

New Delhi metallo-β-lactamase (*bla*_NDM_) was first described in 2008 in a Swedish traveler returning from the Indian subcontinent [10]. Since then, *bla*_NDM_ has been documented in all continents, with the earliest *bla*_NDM_ archived sample from 2005 [11]. Compared with other carbapenemases, *bla*_NDM_ spread is characterized by alarming public health features, including: (1) broad Gram-negative bacterial host range, including highly-virulent bacteria such as *Vibrio cholera* and *Shigella boydii* [12]; (2) frequent acquisition among *Escherichia coli* and *Klebsiella pneumoniae*, which are Gram-negative species carried as gut flora and able to survive in the inanimate environment; (3) widespread presence in the Indian subcontinent, Southeast and East Asia, home to a large proportion of the global human population; and (4) co-carriage with other resistance genes on the *bla*_NDM_-bearing plasmid [13].

Multiple seminal investigations have focused on determining international and local transmission patterns of chromosome-mediated antimicrobial resistance [14–16]. However, there remained many unanswered questions concerning the spread of plasmid-borne antimicrobial resistant genes. While mass global travel and widespread antibiotic use have been widely recognized as population risk factors associated with the dispersal of *bla*_NDM_ [13], little is known regarding the genomic factors associated with its rapid spread [17]. Antimicrobial resistance genes are often carried by mobile genetic elements like plasmids and transposons [18], which may also carry integrons or other gene mobilization elements [19, 20]. A key biologic challenge in understanding plasmid-borne gene molecular epidemiology is its capability to exploit three tiers of gene spread: (1) inter-plasmid gene module transposition; (2) inter-bacterial plasmid conjugation; and (3) bacterial spread among humans, animals and the environment [13]. While single nucleotide polymorphism (SNP)-based phylogenetic methods have been successful in understanding the transmission of chromosome-mediated antimicrobial resistance, these methods are ill-suited to determining the dynamics of multi-tiered gene flow of plasmid-mediated antimicrobial resistance due to the lack of conserved genomic regions across the spectrum of diverse plasmids.

By moving beyond conventional SNP-based phylogenetic study to a plasmid clustering approach based on distances measured by the degree of gene sharing and similarity of shared genes between different plasmids, we analyzed a combined collection of all GenBank complete plasmid sequences within Gram-negative bacteria hosts, thus having an unprecedented opportunity to profile the global dissemination of this important resistance gene. A total of 2749 complete plasmid sequences from NCBI GenBank database were included in this study, of which 39 are *bla*_NDM_-positive. This provided a comprehensive description on the distribution and genetic movement of *bla*_NDM_. Moreover, in order to investigate local transmission of *bla*_NDM_, we sequenced 11 *bla*_NDM_-positive CPE isolates in our institute [21], of which transmission pattern was inferred based on the identity of *bla*_NDM_-positive plasmids and phylogenetic study of the chromosomes, in combination with the patients’ records. In summary, our study suggested that *bla*_NDM_-positive plasmid diversity is very low in a local Singapore hospital context. Analyzing the 11 *bla*_NDM_-positive CPE isolated from the hospital showed that *bla*_NDM_ spread was predominantly characterized by plasmid conjugation and bacterial transmission. In contrast, the *bla*_NDM_-positive plasmid is highly variable in global setting, due to the transposition of the *bla*_NDM_ gene cassette into different plasmids. Furthermore, our analysis revealed that *bla*_NDM_-positive plasmids worldwide can be further clustered into 7 distinct clusters correlated with plasmid incompatibility group and geographical distribution. Our findings advance understanding of plasmid-mediated antimicrobial resistance spread both locally and globally.

## Methods

### Clinical isolates

Tan Tock Seng Hospital (TTSH) is Singapore’s second largest acute-care hospital with 36 clinical and allied health departments and more than 1400 beds. The first case of carbapenemase-producing *Enterobacteriaceae* (CPE) in TTSH was detected in September 2010 (subject 16). Following the index case, CPE was detected in another 7 patients. Among these 7 patients, 2 shared a ward with the index patient during time at risk (ie, epidemiologically linked contacts), while the other 5 were detected via their clinical cultures. All the *Enterobacteriaceae* isolates from these CPE-positive patients were found to harbor *bla*_NDM_. The infection control response to a new *bla*_NDM_-positive patient detected in the course of routine testing included strict isolation of the index patient, contact tracing within the same ward and in previously admitted wards, and screening of these contacts with rectal swabs for CPE carriage using draft guidelines issued by CDC. Age, gender, travel history, history of ward locations and clinical diagnoses were collected by retrospective case-chart review.

In this hospital laboratory, species identification of *Enterobacteriaceae* was done using matrix-assisted laser desorption ionization–time of flight mass spectrometry (Bruker Daltoniks, Germany) and initial susceptibility testing was performed with VITEK 2 automated system (bioMérieux Vitek, Inc., USA) or using Kirby-Bauer method; susceptibility was defined according to the breakpoints of the Clinical and Laboratory Standard Institute (CLSI). Additional Minimum Inhibitory Concentration (MIC) testing using E-test strips (AB bioMerieux, Sweden) was done for all *Enterobateriaceae* isolates which were found to be non-susceptible to imipenem or meropenem on Mueller-Hinton plates; those isolates with a MIC ≥ 2 mgs/L were investigated further. Characterization of β-lactamase genes was performed by a polymerase chain reaction (PCR) assay targeting three carbapenemases with potential for rapid spread: *bla*_NDM-1_ [22], *bla*_KPC_ [23] and *bla*_OXA-48_-like [24].

### DNA sequencing and genome assembly of clinical isolates

Whole genome sequencing was performed on the Illumina MiSeq platform (Illumina Inc., USA) as detailed before [21]. Sequence reads have been submitted to the European Nucleotide Archive (ENA) under accession PRJEB13304. *De novo* assembly was performed using Velvet [25], parameters of which were optimized by VelvetOptimiser (http://bioinformatics.net.au/software.velvetoptimiser.shtml) with k-mer lengths ranging from 55 to 63. For all of the 11 isolates, VelvetOptimizer achieved the best assembly at the k-mer length of 63.

Species identification for each isolate was performed using BLAST to search the assembled contigs in NCBI nt database. If the top five BLAST results for a contig are from chromosomal DNA, these chromosomal genomes would be considered as candidate chromosomes for the isolate. For each isolate, each candidate chromosomes would be used as the reference sequence, against which all the contigs would be aligned. The genome coverage by the contigs would then be calculated, where the candidate chromosome with the highest genome coverage would be taken as the most similar bacterial strain and its species would be identified as the bacterial species of the isolate. Multi-locus sequence type (MLST) databases for *E. coli* (Achtman scheme) and *K. pneumonia* were downloaded from pubmlst.org [26], BMC Bioinformatics and the assembled contigs were search against the gene alleles to infer MLST.

### Molecular epidemiology

Sequence reads were aligned to the reference genome (JJ1886 [GenBank: CP006784.1] for *E. coli*, and HS11286 [GenBank: CP003200.1] for *K. pneumoniae*) using BWA [27]. Single-nucleotide variants were called using SAMtools [28]. Positions with less than 10 reads or with a minor allele frequency larger than 0.25 would be marked as ‘unknown’ data. Variants would then be called if the alternate allele frequency is above 0.75. Maximum likelihood phylogenetic trees were constructed using RAxML [29] where a substitution model of GTRGAMMA was used and rapid bootstrap analysis was conducted on 500 runs. Neighbor-Joining trees were constructed using MEGA6 [30], where evolutionary distances were computed using the Kimura 2-parameter method and bootstrap test was conducted on 500 replicates.

### *bla*_NDM_-positive plasmid identification

For each isolate, the contig with the *bla*_NDM_ gene was first identified and extracted, after which the contig sequence was searched in the NCBI nt database for complete plasmid sequences with more than 2000 base-pair identity. The similar complete plasmid sequences were then each used as the reference genome, against which all the contigs were aligned to calculate the sequence coverage by the contigs. The complete sequences with the highest sequence coverage would then be taken as the most similar plasmids.

### Plasmid mapping, genome coverage calculation and variant calling

Novoalign was used for reads mapping against a reference plasmid sequence, after which indel realignment was conducted with GATK IndelRealigner [31], and the coverage was calculated with GATK DepthOfCoverage. Variants were called with UnifiedGenotyper in GATK, with filtering criteria: ″MQ < 40.0, QD < 2.0, FS > 60.0, HaplotypeScore > 13.0″.

### Conjugation assay

Conjugation assays were performed using each of the 11 clinical isolates and an azide-resistant *E. coli* J53 as previously described [32]. To test the possibility of in vivo horizontal gene transfer in subject 21, conjugation assay was repeated by mixing donor *K. pneumoniae* isolates (KP-U-141010 and KP-R-141010) with the recipient strain (EC-R-141010). *E. coli* trans-conjugants were selected using selective agar (Brilliance UTI Agar, Eosin methylene blue agar) supplemented with meropenem (10 mg/L).

### Complete plasmid sequences

All the 2749 available complete plasmid sequences within Gram-negative bacterial host in the NCBI plasmid database (April 2014) were downloaded for analysis, of which 39 are *bla*_NDM_-positive. Information on sampling location and date, sample source, subject’s travel history, host bacterial species and bacterial anti-microbial resistance phenotypes were obtained from GenBank entries or accompanying peer-reviewed references cited in GenBank.

### Plasmid clustering

The plasmid clustering was conducted based on the virtual hybridization method as described by Zhou et al. [33] to investigate the similarity of the diverse complete plasmid sequences.

For each plasmid, all coding DNA sequences, as determined by original investigators, were downloaded from NCBI. Duplicate genes on the same plasmid, defined as coding DNA sequences having similarity value [similarity value = length of matching sequences)*(BLAST identity)/(length of reference sequence)] above 0.45, were removed. This resulted in a set of 234,450 genes. Additionally, insertion sequences within each plasmid were detected using IS Finder (https://www-is.biotoul.fr/) with default parameters at a cut-off e-value of 1e^−20^, which identified 1496 unique insertion sequences.

For genetic sequence comparison, a similarity score is calculated as 2*(length of matching sequences)*(BLAST identity)/(length of reference sequence + length of matching sequences). The 2749 complete plasmid sequences were then compared using nucleotide BLAST algorithm against each of the 234,450 genes and 1496 insertion sequences to calculate a similarity score, which resulted in a 2749 by 235,946 matrix of similarity scores. A hypothetical plasmid sequence with all similarity scores set to zero was used as outgroup.

To achieve computational tractability, 1000 random matrices were generated, each of which was composed of 20 % of the similarity score matrix’s columns that were randomly selected without replacement, showing the similarity scores represented by the 20 % of the randomly selected genes or insertion sequences. For each matrix of similarity scores, pair-wise Euclidean distances between plasmid sequences were calculated and formulated into a distance matrix, after which a Neighbor-Joining tree was constructed with the ‘neighbor’ program in PHYLIP [34]. A consensus tree was constructed using the ‘consense’ program in PHYLIP with the majority rule as the consensus type.

*bla*_NDM_-positive plasmid clusters based on the consensus tree were defined using a stringent criterion of at least 2 unique *bla*_NDM_-positive plasmids, with all nodes having ≥99 % support at 1000 bootstraps.

### Phylogenetic tree for cluster refinement

Cluster refinement was conducted for each cluster respectively. For each cluster, coding DNA sequences present in all plasmid sequences with a nucleotide BLAST e-value less than 1e^−5^ and an identity above 80 % were extracted, aligned and concatenated. The phylogenetic trees were built for each cluster similarly as in the molecular epidemiology study.

### Incompatibility groups of plasmids

Plasmid incompatibility (Inc) groups were assessed using PlasmidFinder from the Center for Genomic Epidemiology (CGE) with default settings [35].

### Comparative genomics

To compare the plasmids, complete plasmid sequences and their corresponding gene features were first downloaded from NCBI Genbank. The annotated plasmid sequences were then compared and visualized with the Artemis comparison tool ACT [36].

## Results

### Local *bla*_NDM_-positive plasmid diversity in a single hospital

**Fig. 1:**
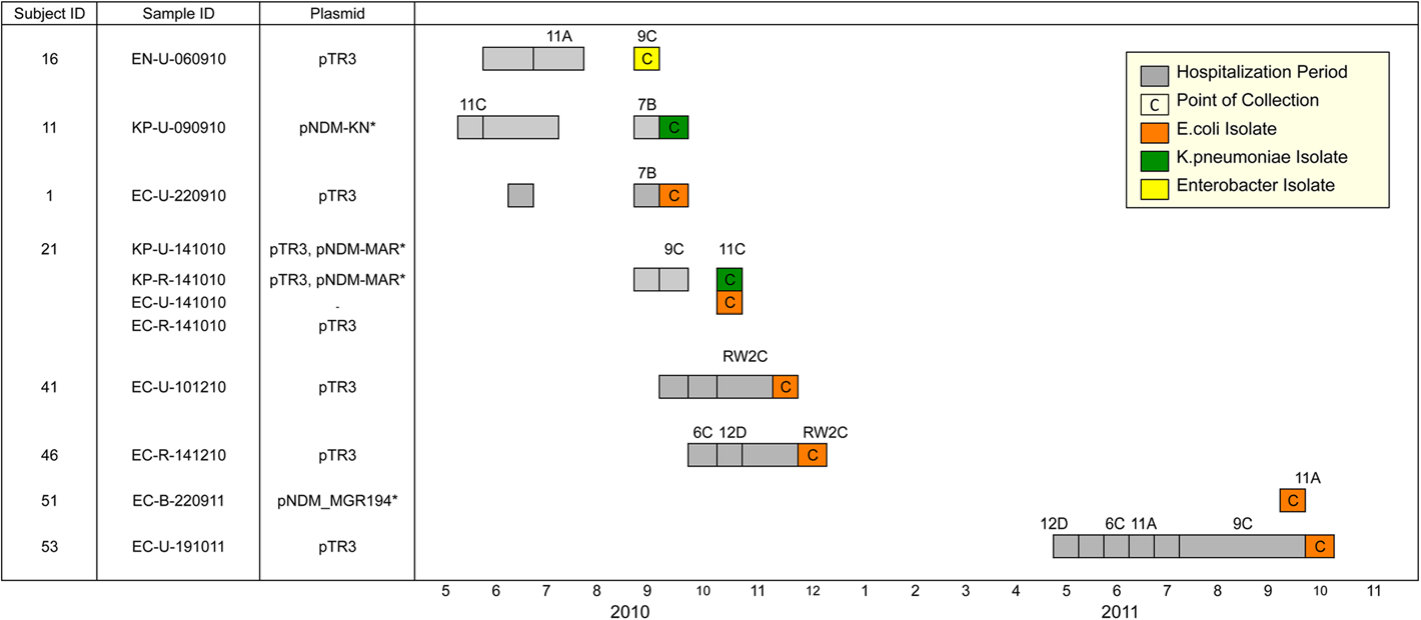
Patient transmission dynamics of local bacteria samples. The *bla*_NDM_ cases were identified in a local hospital from 2010 to 2011 as represented in the timeline. Each patient is represented by a horizontal track. Subject ID, sample ID and *bla*_NDM_-positive plasmid found in the isolate are indicated in the first 3 columns. Patient’s stay in the same ward is denoted by gray box. Only wards with ≥2 reported *bla*_NDM_ cases are indicated in the diagram. *: closest reference plasmid identified based on minimum 75 % sequence coverage

The first 11 CPE isolates from 8 patients in a single Singapore hospital were isolated, of which the patient demographics and sample features are summarized in Table 1 and Fig. 1. The median duration of hospitalization to positive CPE culture was 3 days (range: 1–153 days). Six patients (subjects 16, 11, 1, 41, 51 and 53) had *bla*_NDM_ detected on clinical cultures. One patient (subject 21) was co-infected with 4 CPE isolates, where 2 different strains of *Enterobacteriaceae* were isolated from the patient’s stool and urine samples, respectively. Of the 8 patients, only two had travelled out of Singapore in the past 2 years, including subject 21, who had travelled to Australia and subject 41, who had travelled to Malaysia. Whole genome sequencing was conducted on Illumina MiSeq, with the sequencing statistics summarized in Additional file 1: Table S1.

**Table 1:** Patient demographics and sample features

Subject ID	Travel history	Clinical diagnosis	Sample ID	Rationale for sample	Identity of NDM encoding plasmid	MLST
16	NA	Colonization	EN-U-060910	Clinical Sample	pTR3	NA
11	NA	Disease	KP-U-090910	Clinical Sample	pNDM-KN^a^	437
1	NA	Colonization	EC-U-220910	Clinical Sample	pTR3	410
21	Australia	Colonization	KP-U-141010	Clinical Sample	pTR3	48
		Colonization	KP-R-141010	Clinical Sample	pTR3	48
		Colonization	EC-U-141010	Clinical Sample	NA	69
		Colonization	EC-R-141010	Clinical Sample	pTR3	69
41	Malaysia	Colonization	EC-U-101210	Clinical Sample	pTR3	131
46	NA	Colonization	EC-R-141210	Contact Screening for Index Subject 41	pTR3	131
51	NA	Disease	EC-B-220911	Clinical Sample	pNDM_MGR194^a^	205
53	NA	Disease	EC-U-191011	Clinical Sample	pTR3	131

Sample ID format: Organism- Specimen site-Date of Isolation (DD/MM/YY)

Organism: EC = *Escherichia coli*, KP = *Klebsiella pneumoniae*, EN = *Enterobacter cloacae*

Specimen site: U = Urine, R = Rectal swab, B = Bile

^a^Closest reference plasmid identified based on minimum 75 % reference sequence coverage

Plasmid identification was conducted with *de novo* assembly in combination with *bla*_NDM_-positive plasmid identification, plasmid mapping and genome coverage calculation as elaborated in the Methods. The *de novo* assembly statistics were summarized in Additional file 1: Table S1. Among the 11 samples, 10 *bla*_NDM_-positive plasmids were identified, of which 8 were identified as pTR3 [GenBank:JQ349086], 1 was identified as pNDM-KN [GenBank:NC_019153] with the last being identified as pNDM_MGR194 [GenBank:NC_022740] (Table 1). Plasmid identification was most confident for the 41,187 base-pair plasmid pTR3 (100 % genome coverage in all the 8 identified samples at very high read depths) and the 46,253 base-pair plasmid pNDM_MGR194 (100 % genome coverage in sample EC- B-220911 at reasonable read depths). The 162,746 base-pair plasmid pNDM-KN was identified in sample KP-U-090910 with 76.3 % genome coverage at very high read depths. No *bla*_NDM_-positive plasmid was detected in sample EC-U-141010. The genome coverages and the read depths were summarized in Additional file 1: Figure S1 and Table S2, respectively.

Variant calling was performed for the 8 samples containing pTR3, the most prevalent *bla*_NDM_-positive plasmid, to compare the pTR3 plasmid sequences in respective samples with the reference pTR3 sequence [GenBank:JQ349086]. Inspection of the variants revealed that 7 pTR3 plasmid sequences were identical to the reference pTR3 sequence, while one pTR3 plasmid sequence had only one SNP compared to the reference pTR3 sequence. In EN-U-060910 (isolated from subject 16), the pTR3 sequence had one synonymous mutation at the coding region of a putative transposase (position 22107), resulting a codon change of GCC → GCT.

These results showed that local *bla*_NDM_-positive plasmids had limited diversity with the majority of the plasmids being identical copies of pTR3, which is a strong indication of clonal plasmid spread. The other two *bla*_NDM_-positive plasmids had identities of pNDM-KN and pNDM_MGR194. The major difference between the three plasmids (pTR3, pNDM-KN and pNDM_MGR194) strongly indicated independent plasmid introductions into the hospital ecology.

### Bacterial host range at the local level

The bacterial species harboring *bla*_NDM_-positive plasmids were: *E. coli* (7/11), *K. pneumoniae* (3/11) and *Enterobacter cloacae* (*E.cloacae,* 1/11) (Table 1). Of the 7 *E. coli* isolates, 3 were most similar to ST131 *E. coli* strain NA114 [GenBank:NC_017644], while the remaining isolates were most similar to ST23 *E. coli* strain APEC O78 [GenBank:NC_020163], ST597 strain UMN026 [GenBank:NC_011751] and ST1128 strain IAI1 [GenBank:NC_011741]. For the *K. pneumoniae* isolates, three *K. pneumoniae* strains were identified to be similar, including: ST11 strain HS11286 [GenBank:NC_016845], ST23 strain NTUH-K2044 [GenBank:NC_012731] and ST23 strain 1084 [GenBank:NC_018522]. Consistent with previous report [37], there appeared to be no evidence of association between *Enterobacteriaceae* host species and specific plasmid identities.

**Fig. 2:**
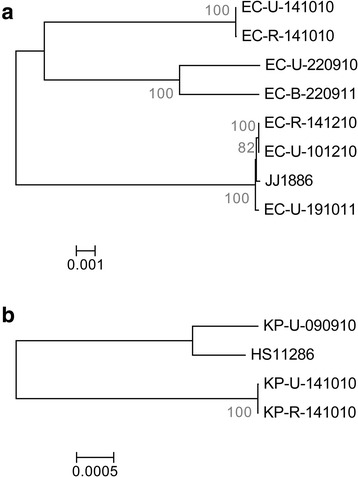
Whole-genome phylogenetic tree of local *bla*_NDM_-positive bacteria. Maximum likelihood trees were constructed based on sequence alignments of *E. coli* (**a**) and *K. pneumoniae* (**b**). JJ1886 and HS11286 are the reference genomes for *E. coli* and *K. pneumoniae*, respectively. The branch lengths were calculated by RAxML and reflect the number of expected mutations per site. Bootstrap values are in a scale of 0–100, and are shown at each node in *grey*

Maximum likelihood phylogenetic trees were constructed for bacteria chromosomes respectively for *E. coli* (Fig. 2a) and *K. pneumoniae* (Fig. 2b), and were supported by Neighbor-Joining trees in Additional file 1: Figure S2*.* Phylogenetic trees for both *E. coli* and *K. pneumoniae* displayed significant diversity. The diversity of bacterial strains harboring pTR3 highlighted the propensity of *bla*_NDM_-positive plasmids to spread via inter-bacterial plasmid conjugation, and would explain a key challenge in relying upon bacterial phylogenetic analysis alone to understand *bla*_NDM_ dissemination.

### Inter- and intra- patient bacterial spread at the local level

Phylogenetic trees of the bacteria chromosomes in Fig. 2 suggested clonal bacterial spread in 3 instances. The first instance involved ST131 *E. coli* detected in 2 patients – subjects 41 and 46, which clustered tightly as EC-U-101210 and EC-R-141210 in Fig. 2a and differs by only 4 SNPs. The limited number of SNPs identified thereby suggested inter-patient bacterial spread.

The other two instances involved bacteria with identical sequence types isolated from different body sites in the same patient (subject 21). KP-U-141010 (isolated from urine) and KP-R-141010 (isolated from rectal swab) were both ST48 *K. pneumoniae* that harbored the pTR3 plasmid, which clustered tightly in Fig. 2b with 25 SNPs. EC-U-141010 (isolated from urine) and EC-R-141010 (isolated from rectal swab) are both ST69 *E. coli* that clustered tightly in Fig. 2a with 58 SNPs. Sample EC-U-141010 was *bla*_NDM_-negative and positive for *bla*_IMP-1_, a class B carbapenemase. In vitro trans-conjugation assays demonstrated the ability of pTR3 to conjugate from *K. pneumoniae* to *E. coli*. Mating assays with pTR3-positive *K. pneumoniae* from subject 21 as the donor and azide-resistant *E. coli* J53 as the recipient demonstrated pTR3 plasmid was self-conjugative (Additional file 1: Figure S3). Subject 21, as a result, represents a possible case of intra-host conjugation.

As discussed, the pTR3 plasmids remained 100 % identical in all but 1 isolate at the nucleotide level in scenarios of inter- and intra-patient bacterial transfer, and inter-bacterial plasmid conjugation within the same host. These results suggested early spread of endemic plasmids at the local level was predominantly clonal.

### Clustering of global plasmids from Gram-negative bacterial host

Complete genomic sequences of 2749 plasmids within Gram-negative bacterial hosts were downloaded from the NCBI database. The median plasmid sequence length is 30,949 base-pairs (range: 744–2,580,084), with the median number of genes annotated per plasmid being 36 (range: 1–2235). Out of the 2749 plasmids, the majority belong to the *Enterobacteriaceae* family (*n* = 877, 31.9 %), followed by *Spirochaetaceae* (*n* = 405, 14.7 %), *Rhodobacteraceae* (*n* = 85, 3.1 %), *Moraxellaceae* (*n* = 81, 2.9 %), and others (*n* = 1301, 47.3 %).

Out of the 2749 plasmid sequences belonging to Gram-negative bacteria, 39 sequences were found to be *bla*_NDM_-positive (Additional file 1: Table S3). These plasmids were sampled from all continents except Antarctica over an 8 year period (2005–2013). Thirty-eight of the 39 *bla*_NDM_-positive plasmid samples have a human origin, while one sample has an environmental origin. The median plasmid sequence length for *bla*_NDM_-positive plasmids is 73,209 base-pairs (range: 35,947–288,920), with the median number of genes annotated per plasmid being 89 (range: 31–372).

**Fig. 3:**
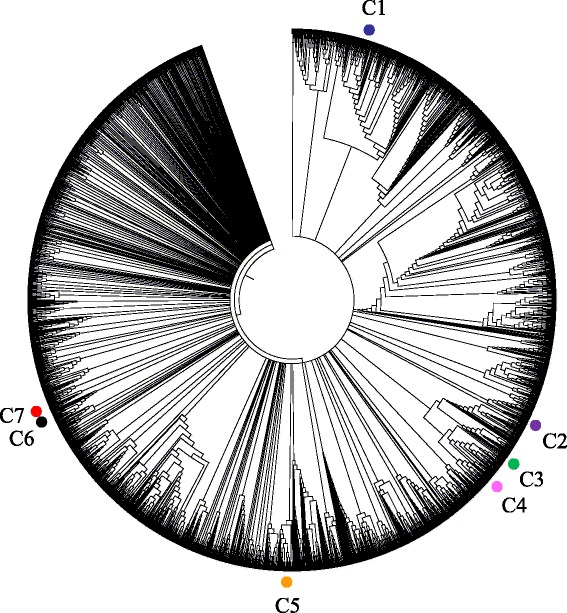
Clustering of global plasmids in Gram-negative bacteria hosts. The Neighbor-Joining tree consisting of 2749 Gram-negative plasmid sequences was constructed to reflect the gene composition similarity of the plasmids. Seven *bla*_NDM_-positive plasmid phylogenetic clusters were identified using stringent criteria (all nodes ≥99 % bootstrap support, minimum 2 unique *bla*_NDM_-positive plasmids). Clusters with *bla*_NDM_-positive plasmids are indicated with dots that are colored distinctively with *blue* (C1), *purple* (C2), *green* (C3), *magenta* (C4), *orange* (C5), *grey* (C6) and *red* (C7)

While construction of a SNP-based phylogenetic tree is the most common method to investigate evolutionary relationships among groups of organisms or strains, it is not applicable to plasmid phylogenetic study as there is no common genomic region shared among all the 2749 complete plasmid sequences. An alternative approach based on the relative distances measured by the degree of gene sharing and the similarity of shared genes has been applied to cluster the plasmids. The pair-wise distances based on a total of 234,450 genes and 1496 insertion sequences present in at least 1 plasmid were calculated as elaborated in the Methods, resulting in a Euclidean-distance derived distance matrix. A Neighbor-Joining tree was constructed with the distance matrix, upon which clustering analyses were based (Fig. 3). The clustering of global plasmid showed high global plasmid diversity with *bla*_NDM_-positive plasmids located in different clusters.

### Clustering and phylogenetic study of *bla*_NDM_-positive plasmids

**Fig. 4:**
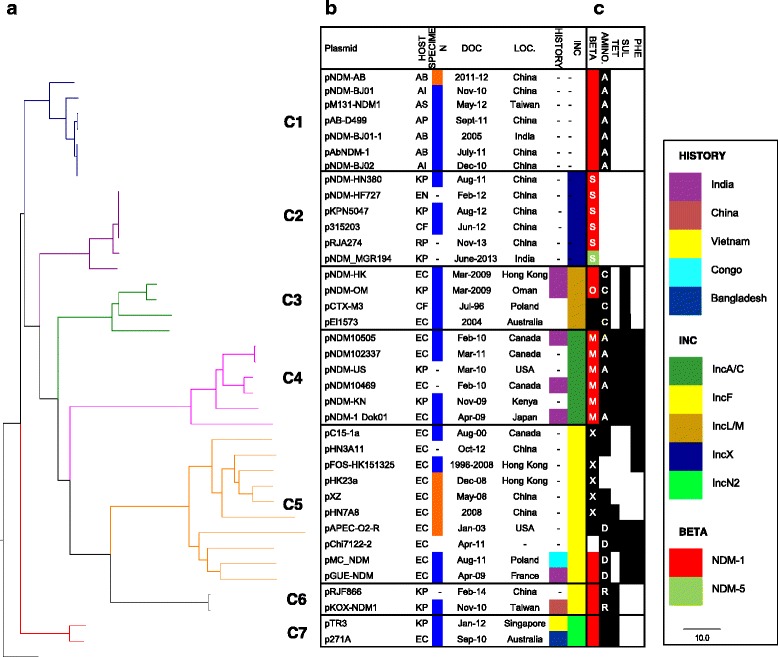
Clustering of *bla*_NDM_-positive plasmids. **a** Neighbor-Joining tree of the 7 *bla*_NDM_ clusters. Branches of each cluster are colored according to the same color scheme as Fig. 3. The tree is rooted using outgroup in black. Branch lengths were Euclidean distances calculated from similarity scores and are reflective of the similarity of plasmid gene composition and the similarity of shared genes. **b** Table showing the identity (PLASMID), bacterial host (HOST), specimen type (SPECIMEN), date of collection (DOC), geographical sampling location (LOC), travel history (HISTORY) and incompatibility group (INC) for each plasmid. Abbreviations: AB, *Acinetobacter baumannii*; AI, *A.iwoffii*; AP, *A. pittii*; AS, *A. soli*; CF, *Citrobacter freundii*, EN, *Enterobacter cloacae*; EC, *Escherichia coli*; KP, *Klebsiella pneumoniae*; RP, *Roultella planticola*. **c** The matrix displays the resistance genetic determinants identified in the corresponding plasmid genome. A *black-shaded* box indicates a positive genotypic trait conferring resistances, the antibiotic classes of which are indicated by the text at the top of the column. Resistance determinants against the following antibiotics were identified: β-lactam, BETA; aminoglycoside, AMINO; tetracycline, TET; sulphonamide; SUL; phenicol; PHE. Abbreviations: A, APH; C, AAC; D, AAD; K, KPC; M, CMY; O, OXA; S, SHV; R, RMT; X, CTX. Presence of *bla*_NDM-1_ was *shaded red* and *bla*_NDM-5_ *shaded green*

Seven clusters (represented by red dots in Fig. 3) were identified to contain *bla*_NDM_-positive plasmids, which range in size from 2 to 10 plasmids. For better clarity, the plasmids within the seven clusters and the clusters near the seven clusters were extracted to construct a new Neighbor-Joining tree with the plasmids’ information summarized (Additional file 1: Figure S4). The plasmids within the seven clusters were extracted and a new Neighbor-Joining tree was constructed, which is presented as Fig. 4 with the plasmids’ information.

The number of shared regions increased markedly for plasmids within the same cluster, allowing for the construction of a phylogenetic tree based on nucleotide sequence alignment within the shared regions. For clusters with more than three sequences, a concatenated alignment of the homologous sequences was generated, after which a phylogenetic tree would be constructed to study the phylogenetic relationship (Additional file 1: Figures S5 and S6). The concatenated sequences within each cluster showed great similarity to each other, as can be identified by the short branch lengths.

While the distance-based clustering method provided a tree based on the gene composition similarity, the cluster refinement phylogenetic tree used SNPs to investigate the evolutionary relationship within each cluster, which were similar in topology with the clustering method.

### Global *bla*_NDM_-positive plasmid diversity: gene transposition

**Fig. 5:**
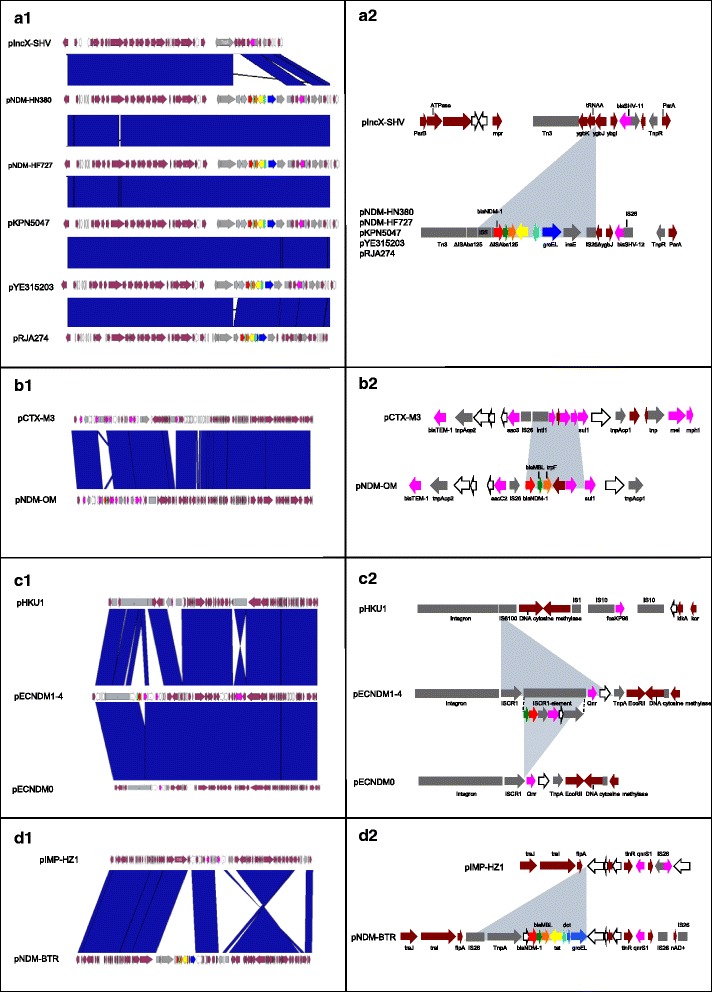
Acquisition of *bla*_NDM_ cassettes. A1, B1, C1 and D1: A comparison of the *bla*_NDM_-positive plasmid genomes with their putative backbone plasmids as identified in the plasmid clustering. The corresponding backbone plasmids are placed at the top of each column. *Blue bands* between panels indicate nucleotide BLAST matches with more than 99 % sequence similarity. A2, B2, C2 and D2: Schematic representations of insertions in the *bla*_NDM_-positive plasmids (*shaded in light blue*) corresponding to A1, B1, C1 and D1. Annotated genes in these regions are colour coded. *Arrows* indicate predicted open-read frames (ORFs), genes with known functions (*maroon*), antimicrobial resistance genes (*magenta*), transpositional genetic elements (*gray*) and hypothetical proteins (*white*). Genes from the *bla*_NDM_ cassette are indicated by arrows coloured as follows: red, *bla*_NDM_; *green*, *ble*_*MBL*_; *orange*, *trpF*; *yellow*, *tat*; *light blue*, *dct*; and *dark blue*, the *groES- groEL* cluster. Plasmid pECNDM represents an NDM-negative laboratory-derived plasmid, where the NDM cassette was mobilized from pECNDM1-4 as a free form

At least 6 events in the 7 clusters (C1–C7) of *bla*_NDM_-positive plasmids have been observed to indicate independent recombination events introducing *bla*_NDM_ into different plasmid backbones of *bla*_NDM_-negative plasmids with subsequent evolution and spread (Fig. 5 and Additional file 1: Figure S7).

In the process of adaptive evolution, diversity of the microbial genome is primarily driven by recombination or point mutations [38, 39]. As the clustering approach made use of plasmid gene composition diversity arising through recombination rather than point mutations, our findings suggested the *bla*_NDM_-positive plasmids have undergone extensive mobile genetic element transposition to adapt to varying environmental niches. As mentioned earlier, there was minimal intra-cluster SNP difference, suggesting that polymorphisms due to point mutations play minimal role to account for the diversity of the plasmids.

Transpositions facilitated by transposons (Tn), insertion sequences (IS) elements and IS common region (ISCR) are detected frequently in plasmids that involve antimicrobial genes, non-antimicrobial genes and transposable genetic elements. With respect to the *bla*_NDM_ gene, transposition mechanism involving *bla*_NDM_ was discernible by comparative genomics in 4 instances: pNDM_HN380 [GenBank: JX104760] (C2, IS*Aba125-*mediated transposition, Fig. 5a), pNDM-OM [GenBank: JX988621] (C3, recombination into Tn1548-borne class I integrin, Fig. 5b), pEcNDM [GenBank: NC_023909] (unclustered, IS*CR1*-mediated transposition, Fig. 5c) and pNDM-BTR [GenBank: KF534788] (unclustered, *fip*A gene hotspot recombination, Fig. 5d). The Tn125 composite transposon platform has been theorized to be the original vehicle to mobilize *bla*_NDM_ among *Acinetobacter* species. Our results reveal that *bla*_NDM_ introductions also occurred in the context of IS*CR1*-mediated transposition, *fip*A gene hotspot recombination and Tn1548-borne class I integron recombination. Larger datasets of genomic sequences involving *bla*_NDM_-positive and negative nearest neighbors will enhance the understanding of *bla*_NDM_ transposition globally.

### Global *bla*_NDM_-positive plasmid diversity: incompatibility group and geographical distribution

The plasmid clustering based on gene composition diversity tends to cluster the plasmids with the same backbone together, thus showing a clear clustering of the plasmid Inc groups for *Enterobacteriaceae* plasmids: plasmids in C2 are all Inc X plasmids, plasmids in C3 are Inc L/M, plasmids in C4 are Inc A/C, plasmids in C5 and C6 are Inc F, while plasmids in C7 are Inc NII (Fig. 2).

The plasmid clusters also showed some association with geographical distributions. Some clusters were spreading mainly via regional transmission to date: (1) C1, a cluster of plasmids *Acinetobacter* sp. host, is limited to South Asia and East Asia; (2) C2 and C6 are limited to South and East Asia; and (3) C7 was found in Southeast Asia and Oceania. Other clusters (C3, C4 and C5) had wider geographic dispersion involving South Asia, East Asia, Middle East, North America, Africa and Europe.

### Local *bla*_NDM_-positive plasmid in the global context

As detailed in the global analysis, pTR3 clustered tightly with p271A [GenBank: JF785549], a plasmid described in Australia (Fig. 4, C7). The other two plasmids located in different plasmid clusters: pNDM-KN is in C4, and pNDM_MGR194 is in C2. In contrast to global plasmid diversity, the presence of near identical pTR3 plasmids in 8 out of 11 local samples suggested the *bla*_NDM_-positive plasmid diversity at the local level to be very low. On the other hand, the 2 non-pTR3 plasmids, which were related to different plasmid clusters in the global plasmid phylogeny, were detected each in only one patient, which suggested independent plasmid introductions into the hospital ecology.

## Discussion

By analyzing whole genome sequencing data of 11 *bla*_NDM_-positive CPE isolated in a local hospital and 2749 complete plasmid sequences (including 39 *bla*_NDM_-positive plasmids) in the NCBI database, we investigated the local transmission and global dissemination of the *bla*_NDM_ gene. Our analysis has highlighted the complex genetic pathways of *bla*_NDM_ spread. Globally, *bla*_NDM_ spread involved marked plasmid diversity with no predominant bacterial clone. The *bla*_NDM_-positive plasmids were carried by multiple species of *Acinetobacter* and *Enterobacteriaceae*, thereby highlighting the propensity for conjugation of *bla*_NDM_-positive plasmids among different bacterial species. The *bla*_NDM_ gene module mobilized between different plasmid backbones on at least 6 independent occasions. In contrast to global plasmid diversity, early local spread of *bla*_NDM_-positive plasmids in a single Singapore hospital was characterized by clonal spread of a predominant plasmid pTR3 with 2 sporadic instances of plasmid introduction (pNDM-KN and pNDM_MGR194).

The plasmid clustering approach is crucial to our current analysis as it allows quantitative analyses of plasmid molecular epidemiology involving a large number of diverse plasmids as a tool in analyzing global spread of plasmid-borne genes. Prior genomic investigations of *bla*_NDM_ spread have been mainly restricted to comparisons of less than 10 closely related plasmids due to the lack of phylogenetic congruence, and hence have not been able to discern the patterns of *bla*_NDM_-positive plasmid clustering at a global level. The establishment of nearest-neighbor relationships facilitated the determination of transposition events involving genomic regions (genes and insertion sequences). Determination of cluster relationships subsequently opened the ability to correlate clusters with specific phenotypes (for example, extent of global spread or plasmid Inc groups).

Whole genome studies of successful bacterial clones have been used to understand transmission of chromosomally-mediated antimicrobial resistant bacteria, for example MRSA. However, prior studies relying upon bacteria chromosomes to understand *bla*_NDM_ transmission have been hindered by the diversity of bacterial species and strains harboring *bla*_NDM_, even in a single geographic local [40]. The current study highlighted three vital evolutionary mechanisms underlying *bla*_NDM_-positive bacteria diversity: (1) *bla*_NDM_-gene module transposition, (2) *bla*_NDM_-positive plasmid conjugation, and (3) *bla*_NDM_-positive bacterial spread. Future studies of *bla*_NDM_ transmission would have to take into account these three levels of gene spread.

Gene module transposition was a vital factor in the successful spread of *bla*_NDM_ for at least three reasons: (1) mobilization of *bla*_NDM_ from *Acinetobacter sp.* plasmids to *Enterobacteriaceae* plasmids, as recognized before; (2) mobilization of *bla*_NDM_ among *Enterobacteriaceae* plasmids of differing Inc groups; and (3) non-*bla*_NDM_ gene movement facilitating adaptation of plasmids to differing selection pressures.

It is well accepted that certain plasmid Inc groups can be stably maintained only in closely related bacterial hosts, while others have the ability to replicate in a broader host range [18]. In our study, we noted an association between plasmid Inc groups and geographical spread of the *bla*_NDM_-positive plasmids. Specifically, broad-host-range (BHR) plasmid families (Inc A/C, Inc L/M and Inc F) were associated with transcontinental spread of *bla*_NDM_ in Asia, North America, Africa and Europe. In contrast, narrow-host-range plasmids families (Inc X, Inc FII and Inc NII) had limited spread within the region. This suggested that Inc type contributes to the emergence and global spread of the *bla*_NDM_ gene, as supported by multiple earlier studies. Laboratory experiments have showed that BHR plasmids evolved various strategies that enable them to replicate autonomously in much wider range of phylogenetically distinct hosts [18]. These plasmid types, being well adapted to different bacterial hosts (Inc A/C and Inc L/M), endowed with efficient conjugative system (e.g Inc L/M) promoted the spread of *bla*_NDM_ by enabling its stable maintenance in novel bacterial hosts or ecological niches, often independently from the presence of antimicrobial selection [18]. Collectively, our data suggested that plasmid types, especially those of BHR, play an essential role in plasmid adaptation by improving their stability for long-term persistence in bacterial communities of clinical and natural habitats. A limitation of the global plasmid-based inferences based on the current data is the limited number of samples and potential bias in sample selection, a limitation common for all studies leveraging on public databases. Larger studies with diverse sampling populations would help to address sampling bias.

Local *bla*_NDM_ spread in a single Singapore hospital context was characterized predominantly by conjugation of a clonal plasmid (pTR3) between *Enterobacteriaceae* (inter-bacterial plasmid conjugation), and inter-human host *bla*_NDM-_positive bacterial transmission (bacterial spread). The finding of the pTR3 plasmid in 2 distinct *K. pneumoniae* strains in another Singapore hospital further supported a significant role of inter-human host transmission and clonal plasmid conjugation in local spread. Three recent publications using whole genome sequencing also reported the predominant role of inter-human host transmission (via the inanimate environment in some cases) and horizontal gene transfer in local hospital spread of carbapenemases [40–42].

One potential reason for the difference in the local and the global plasmid diversity is the sampling and the time period. While the 39 global complete *bla*_NDM_-positive plasmid sequences has a long time range of eight years, the 11 local isolates were isolated within a one-year period.

An important limitation of this study and others of its kind is the inevitable bias that comes with selecting isolates. To minimize this inherent limitation, we included all *bla*_NDM_ plasmid sequenced at the point of study, spanning a period of almost 8 years from 2005 to 2014 (note that *bla*_NDM_ was only discovered in year 2009), covering 5 continents. Proportionally, a larger majority (85 %) of the samples came from human origin, while *bla*_NDM_ has been reported to present in livestocks and environment in high prevalence. However, this would not inherently influence the results of this study, that is, linking phenotype (such as antimicrobial resistance, geographical location and host species) with plasmid characteristics (such as *Inc* group).

Our current analysis offers a glimpse of the genetic armamentarium available to *bla*_NDM_ for dissemination to multiple environments. The limited data available for understanding transmission of this important resistance gene is highlighted by availability of only approximately 39 *bla*_NDM_-positive and 2749 Gram-negative whole-plasmid sequences globally. Whole genome sequencing of *bla*_NDM_-positive isolates from diverse geographies on a much larger scale will definitely increase understanding of *bla*_NDM_ evolution and spread, and may prove crucial to long-term control of NDM.

## Conclusions

Our analysis has revealed the complex genetic pathways of *bla*_NDM_ spread, where the global dissemination is mainly characterized by transposition of the *bla*_NDM_ gene cassette into different plasmids while early local transmission is mainly a result of plasmid conjugation and bacterial spread. Our findings advance understanding of plasmid-mediated antimicrobial resistance spread both locally and globally.

## Abbreviations

CPE, carbapenemase-producing Enterobacteriaceae; MLST, multi-locus sequence type; PCR, polymerase chain reaction; SNP, single nucleotide polymorphism.

## Additional file

Additional file 1:
**Figure S1.** Read depths along the reference plasmid sequences based on Illumina MiSeq sequencing reads mapping. **Figure S2.** Whole-genome Neighbor-Joining tree of local *bla*_NDM_-positive bacteria. **Figure S3.** In vitro trans-conjugation assay of local *bla*_NDM_-positive isolates. **Figure S4.** Details of the *bla*_NDM_-positive plasmid clusters. **Figure S5.** SNP-based refinement maximum likelihood trees of *bla*_*NDM*_ plasmid clusters. **Figure S6.** SNP-based refinement Neighbor-Joining trees of *bla*_NDM_ plasmid clusters. **Figure S7.** Investigation of transposition events among *bla*_NDM_ plasmid clusters by recombination analysis. **Table S1.** Summary of Illumina sequencing and *de novo* assembly statistics. **Table S2.** Descriptive statistics for plasmid mapping. **Table S3.** Names and accession numbers of *bla*_NDM_-positive plasmids. **Table S4.** Result summary of recombination analysis. (DOCX 2781 kb)
