# Supplementary material for: Local transmission and global dissemination of New Delhi Metallo-Beta-Lactamase (NDM): a whole genome analysis
Source: BMC Genomics. 2016 Jun 13;17:452. doi: 10.1186/s12864-016-2740-0 (PMC4906610; doi:10.1186/s12864-016-2740-0)
Supplement: Additional file 1: — Figure S1. Read depths along the reference plasmid sequences based on Illumina MiSeq sequencing reads mapping. Figure S2. Whole-genome Neighbor-Joining tree of local bla NDM-positive bacteria. Figure S3. In vitro trans-conjugation assay of local bla NDM-positive isolates. Figure S4. Details of the bla NDM-positive plasmid clusters. Figure S5. SNP-based refinement maximum likelihood trees of bla NDM plasmid clusters. Figure S6. SNP-based refinement Neighbor-Joining trees of bla NDM plasmid clusters. Figure S7. Investigation of transposition events among bla NDM plasmid clusters by recombination analysis. Table S1. Summary of Illumina sequencing and de novo assembly statistics. Table S2. Descriptive statistics for plasmid mapping. Table S3. Names and accession numbers of bla NDM-positive plasmids. Table S4. Result summary of recombination analysis. (DOCX 2781 kb) [file 12864_2016_2740_MOESM1_ESM.docx]

**Supplementary Figures and Tables**

**Supplementary Figure 1. Read depths along the reference plasmid sequences based on Illumina MiSeq sequencing reads mapping**

**Supplementary Figure 2. Whole-genome Neighbor-Joining tree of local *bla*_NDM_-positive bacteria**

**Supplementary Figure 3. *In vitro* trans-conjugation assay of local *bla*_NDM_-positive isolates**

**Supplementary Figure 4. Details of the *bla*_NDM_-positive plasmid clusters**

**Supplementary Figure 5. SNP-based refinement maximum likelihood trees of *bla_NDM_* plasmid clusters**

**Supplementary Figure 6. SNP-based refinement Neighbor-Joining trees of *bla*_NDM_ plasmid clusters**

**Supplementary Figure 7. Investigation of transposition events among *bla*_NDM_ plasmid clusters by recombination analysis**

**Supplementary Table 1. Summary of Illumina sequencing and *de novo* assembly statistics**

**Supplementary Table 2. Descriptive statistics for plasmid mapping**

**Supplementary Table 3. Names and accession numbers of *bla*_NDM_-positive plasmids**

**Supplementary Table 4. Result summary of recombination analysis**


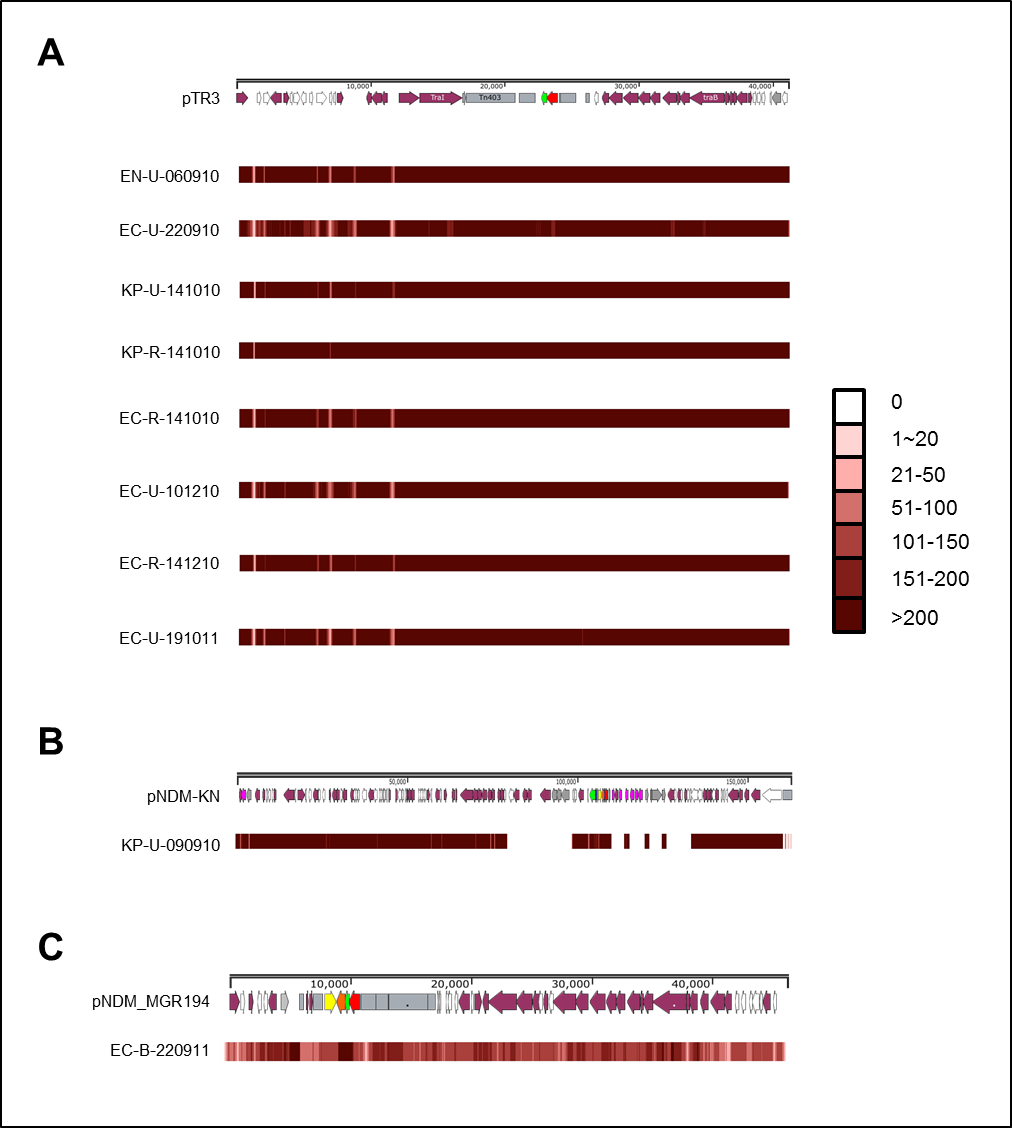


**Supplementary Figure 1. Read depths along the reference plasmid sequences based on Illumina MiSeq sequencing reads mapping.** Sequencing reads were mapped to the plasmid sequences to calculate the read depths along the reference sequences of: pTR3 (A), pNDM­KN (B), and pNDM_MGR194 (C). In A, the read depths are reasonable for all samples along the complete pTR3 sequence, which strongly supports the presence of the pTR3 in the samples. In B, 76% of pNDM-KN has been covered by the sample with reasonable read depths with major absences of genetic sequences. In C, the full length of pNDM_MGR194 has been covered with reasonable read depths, strongly suggests of its presence in the sample.

**
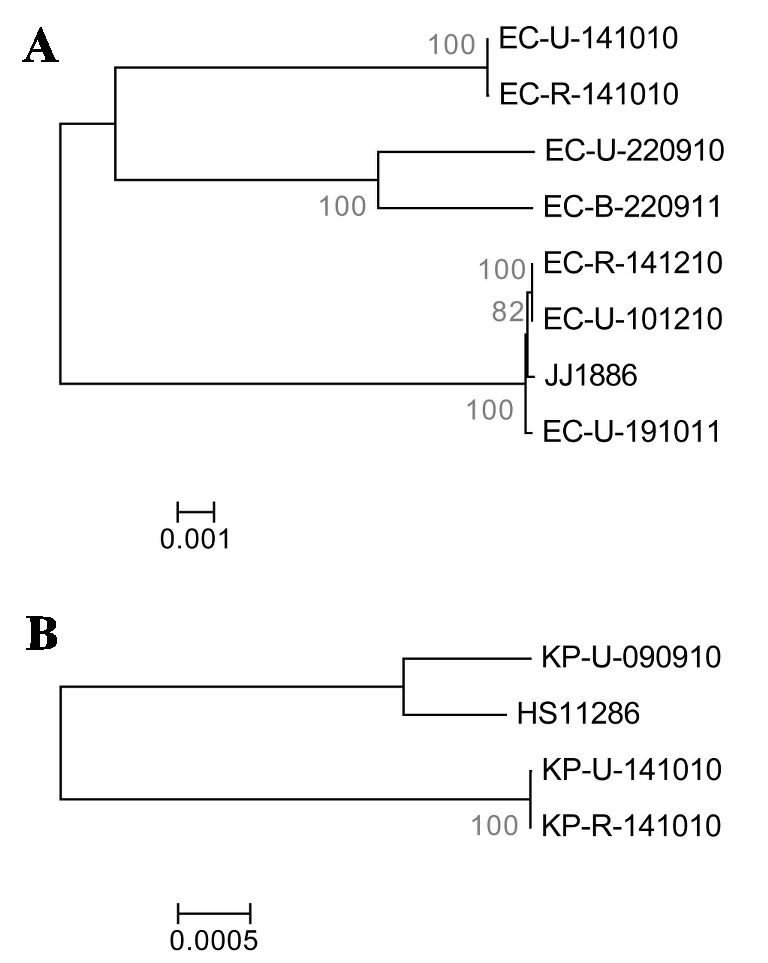
**

**Supplementary Figure 2. Whole-genome Neighbor-Joining tree of local *bla*_NDM_-positive bacteria.** Neighbor-Joining trees were constructed based on sequence alignments of *E. coli* (A) and *K. pneumoniae* (B). JJ1886 and HS11286 are the reference genomes for *E. coli* and *K. pneumoniae*, respectively. The evolutionary distances were computed using the Kimura 2-parameter method and are in the units of the number of base substitutions per site. Bootstrap values are in a scale of 0 to 100, and are shown at each node in grey.


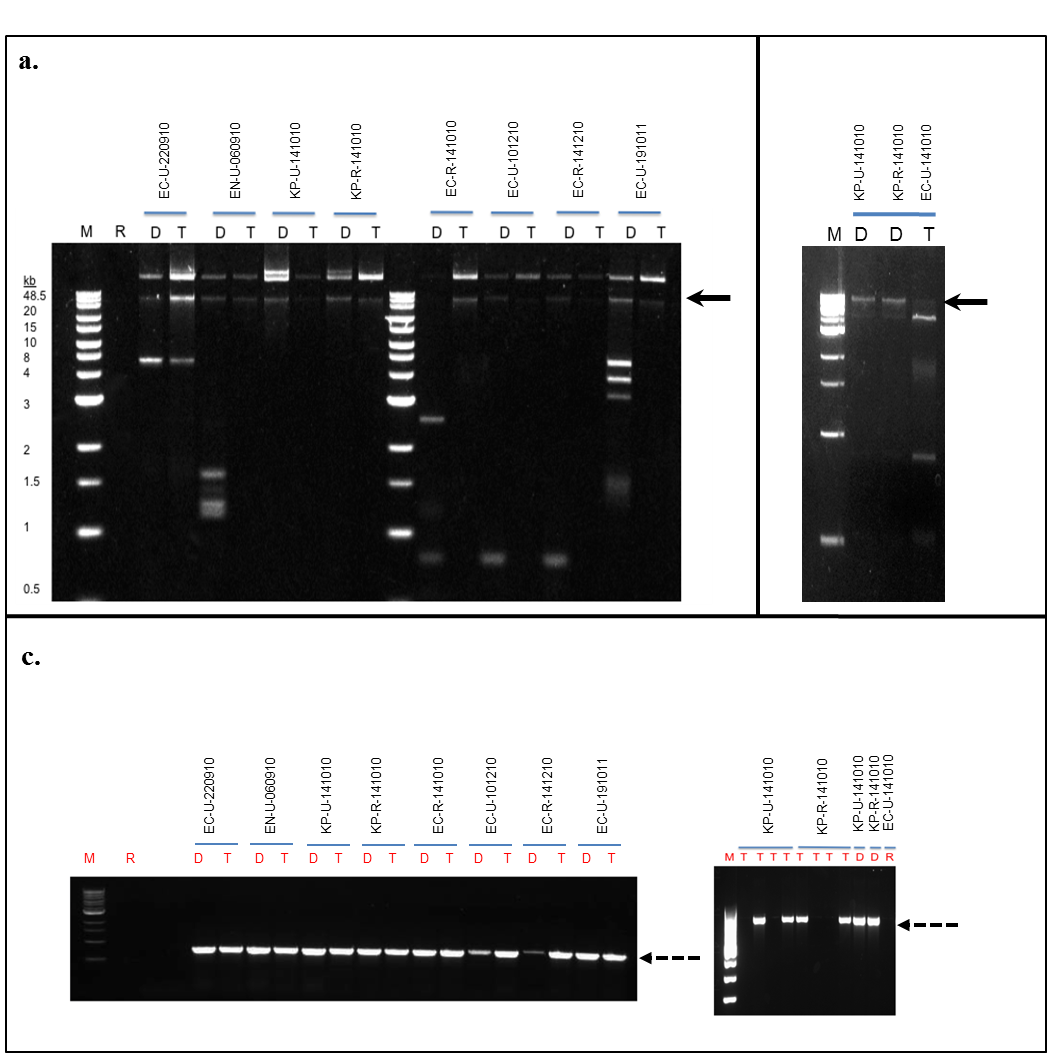


**Supplementary Figure 3. *In vitro* trans-conjugation assay of local *bla*_NDM_-positive isolates.** (A) Trans-conjugation experiments were performed as described in Methods with clinical isolate as the donor (labeled as D) and *E. coli* strain J53 as the recipient (labeled as R). The gel electrophoresis analysis of the donors, recipients and trans-conjugants (labeled as T) are shown. (B) Trans-conjugation experiments were performed with clinical isolates as the donor and an *E.coli* isolate, EC-F86E-U-141010, as the recipient. (C) All donor strains and trans-conjugants were subjected to PCR amplification to detect the presence of the *bla*_NDM_ gene. Solid arrows indicate the expected band size of pTR3. Dotted arrows indicate the expected band size of *bla*_NDM_. (M: DNA marker).

**Figure Legends**

**Supplementary Figure 4. Details of the *bla*_NDM_ plasmid clusters.** On the left: Neighbor-Joining phylogenetic tree, based on which 7 *bla*_NDM_ clusters were defined with the following criteria: most proximal nodes with ≥99% bootstrap support, at least one *bla*_NDM_-positive plasmid. Branches of each cluster are colored distinctively with blue (C1), purple (C2), green (C3), magenta (C4), orange (C5), grey (C6) and red (C7), respectively. On the right: Summary table showing the plasmid identity (PLASMID), bacterial host (HOST), specimen type (SPECIMEN), date of collection (DOC), geographical sampling location (LOC), travel history (HISTORY) and incompatibility group (INC) for each plasmid. The matrix displays the resistance genetic determinants identified in the corresponding plasmid genome. A black-shaded box indicates a positive genotypic trait conferring resistances, the antibiotic classes of which are indicated by the text at the top of the column. A detailed figure legend has been provided at this page.


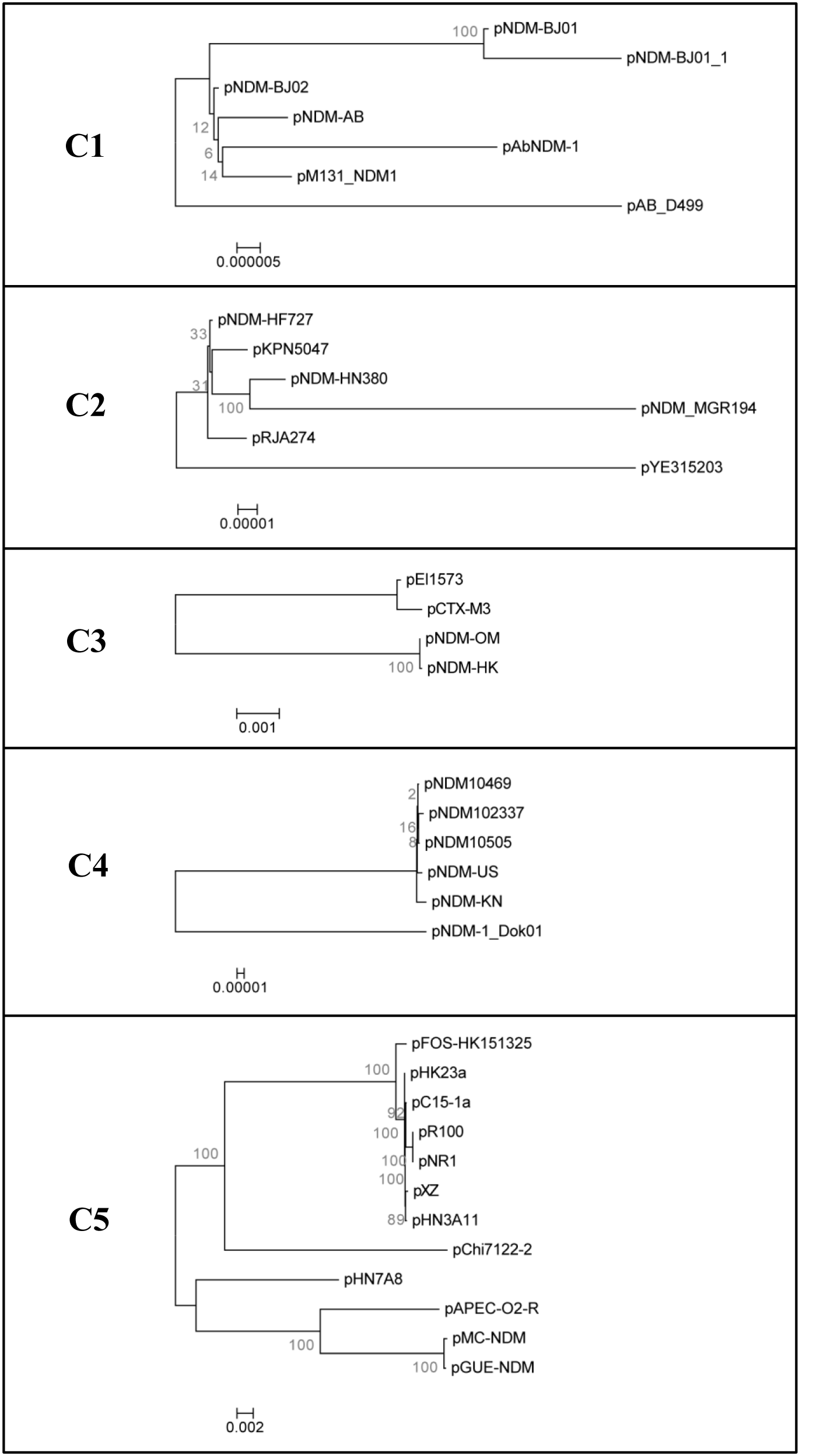


**Supplementary Figure 5. SNP-based refinement maximum likelihood phylogenetic trees of *bla*_NDM_ plasmid clusters.** For each cluster, sequences of all plasmids within this cluster were extracted, whose shared regions were aligned and concatenated for the construction of the maximum likelihood trees shown above. The results for C6 and C7 were not shown as the clusters only consist of 2 isolates each. The branch lengths were calculated by RAxML and reflect the number of expected mutations per site. Bootstrap values are in a scale of 0 to 100, and are shown at each node in grey.

**Supplementary Figure 6. SNP-based refinement Neigobor-Joining trees of *bla*_NDM_ plasmid clusters.** For each cluster, sequences of all plasmids within this cluster were extracted, whose shared regions were aligned and concatenated for the construction of the Neighbor-Joining trees shown above. The results for C6 and C7 were not shown as the clusters only consist of 2 isolates each. The evolutionary distances were computed using the Kimura 2-parameter method and are in the units of the number of base substitutions per site. Bootstrap values are in a scale of 0 to 100, and are shown at each node in grey.

1F:

1E:

1D:

1C:

1B:

1A:

4D:

4G,H:

4E, F:

4A:

4B, C:

pNDM-_Dok01

pNDM-KN

pNDM-US

pNDM10469

pNDM102337

pNDM105

**C1**

**C2**

**C3**

**C4**

**C6**

**C5**

**C7**

**Supplementary Figure 7. Investigation of transposition events among *bla*_NDM_-positive plasmid clusters by recombination analysis.** On the left: A comparison of the different plasmids in each plasmid cluster. Blue bands between the sequences indicated nucleotide BLAST matches with more than 99% sequence similarity. On the right: Schematic representations of genes surrounding the insertion region (shaded in light blue). Annotated genes in these regions are color-coded. Arrows indicate the predicted ORFs with known function (maroon), antimicrobial resistance genes (magenta), transpositional genetic elements (gray) and hypothetical proteins (white). Genes from the NDM cassette are indicated by arrows colored as follows: red: blaNDM, green: bleMBL, orange: trpF, yellow: tat, light blue: dct, and dark blue: the groES-­ groEL cluster. A summary of the results of recombination analysis are included as Supplementary Table 4.

**C7**

**Supplementary Table 1. Summary of Illumina sequencing and *de novo* assembly statistics**

| **Sample ID** | **Illumina sequencing statistics** | | | | ***De novo* assembly statistics** | | | | |
| --- | --- | --- | --- | --- | --- | --- | --- | --- | --- |
|  | **#**  **Reads** | **Reads per pair** | **#**  **Bases** | **Estimated**  **coverage*** | **# Contigs** | **Total**  **length(bp)** | **Maximum**  **length(bp)** | **N50** | **N90** |
| EC-U-220910 | 4638924 | 2319462 | 1159731000 | ~230X | 283 | 4924755 | 311367 | 119021 | 28367 |
| KP-U-090910 | 4993178 | 2496589 | 1248294500 | ~250X | 153 | 5517983 | 679086 | 269381 | 86172 |
| EN-U-060910 | 2551658 | 1275829 | 637914500 | ~125X | 250 | 5360533 | 262681 | 143510 | 35933 |
| KP-U-141010 | 5481114 | 2740557 | 1370278500 | ~275X | 145 | 5628326 | 718541 | 370983 | 84568 |
| KP-R-141010 | 5971648 | 2985824 | 1492912000 | ~300X | 360 | 5847032 | 540865 | 221458 | 27866 |
| EC-U-141010 | 4020020 | 2010010 | 1005005000 | ~200X | 281 | 5471732 | 428602 | 161290 | 30311 |
| EC-R-141010 | 4866162 | 2433081 | 1216540500 | ~245X | 301 | 5515296 | 381553 | 131304 | 26186 |
| EC-U-101210 | 3610924 | 1805462 | 902731000 | ~180X | 248 | 5278528 | 529288 | 172834 | 31299 |
| EC-R-141210 | 3531240 | 1765620 | 882810000 | ~175X | 171 | 5267509 | 452523 | 173849 | 41299 |
| EC-B-220911 | 3694724 | 1847362 | 923681000 | ~185X | 530 | 5238278 | 498227 | 116800 | 22645 |
| EC-U-191011 | 5358750 | 2679375 | 1339687500 | ~270X | 236 | 5314797 | 411061 | 177269 | 25869 |

* Coverage is estimated by Total number of bases (bp)/5,000,000 (bp/genome)

**Supplementary Table 2. Descriptive statistics for plasmid mapping**

| **Sample ID** | **Reference Genome** | | | **# Reads Mapped** | **Genome Coverage** | | **Read Depth** | | | |
| --- | --- | --- | --- | --- | --- | --- | --- | --- | --- | --- |
|  | **Plasmid** | **Accession** | **Length (bp)** |  | **# Sites** | **Percen-**  **tage** | **Mean** | **Medi-an** | **Max** | **Min** |
| EC- U-220910 | pTR3 | JQ349086.2 | 41187 | 96073 | 41187 | 100% | 552.3 | 541 | 1277 | 6 |
| EN- U-060910 | pTR3 | JQ349086.2 | 41187 | 58156 | 41187 | 100% | 335.8 | 268 | 2862 | 2 |
| KP- U-141010 | pTR3 | JQ349086.2 | 41187 | 115116 | 41187 | 100% | 662.5 | 683 | 1234 | 7 |
| KP- R-141010 | pTR3 | JQ349086.2 | 41187 | 190110 | 41187 | 100% | 1098 | 1103 | 1934 | 11 |
| EC- R-141010 | pTR3 | JQ349086.2 | 41187 | 76919 | 41187 | 100% | 443.8 | 438 | 1201 | 4 |
| EC- U-101210 | pTR3 | JQ349086.2 | 41187 | 57693 | 41187 | 100% | 330.5 | 323 | 740 | 0 |
| EC- R-141210 | pTR3 | JQ349086.2 | 41187 | 98697 | 41187 | 100% | 570.8 | 564 | 1102 | 9 |
| EC- U-191011 | pTR3 | JQ349086.2 | 41187 | 69563 | 41187 | 100% | 400.5 | 387 | 1008 | 3 |
| KP- U-090910 | pNDM-KN | NC_019153.1 | 162746 | 169245 | 124099 | 76.30% | 247.8 | 315 | 927 | 0 |
| EC- B-220911 | pNDM_MGR194 | NC_022740.1 | 46253 | 34174 | 46253 | 100% | 170.2 | 133 | 2271 | 2 |

**Supplementary Table 3. Names and accession numbers of *bla*_NDM_-positive plasmids**

| **Name** | **Accession** | **Name** | **Accession** | **Name** | **Accession** |
| --- | --- | --- | --- | --- | --- |
| p271A | JF785549 | pNDM10505 | JF503991 | pNDM-MAR | JN420336 |
| pAB_D499 | NZ_AGFH01000030 | pNDM-1_Dok01 | AP012208 | pNDM-OM | JX988621 |
| pAbNDM-1 | JN377410 | pNDM-1saitama01 | AB759690 | pNDM-US | CP006661 |
| pGUE-NDM | JQ364967 | pNDM-AB | KC503911 | pPrY2001 | KF295828 |
| pKOX_NDM1 | JQ314407 | pNDM-BJ01_1 | JQ001791 | pRJA274 | KF877335 |
| pKp11-42 | KF295829 | pNDM-BJ01 | KF702385 | pRJF866 | KF732966 |
| pKPN5047 | KC311431 | pNDM-BJ02 | JQ060896 | pTR3 | JQ349086 |
| pKPX-1 | AP012055 | pNDM-BTR | KF534788 | pYE315203 | JX254913 |
| pM131_NDM1 | JX072963 | pNDMCFuy | HG428757 | pABCA95 | NC_019322 |
| pMC-NDM | HG003695 | pNDM-CIT | JX182975 | pEcNDM | NC_023909 |
| pMR0211 | JN687470 | pNDM-HK | HQ451074 | pKpNDM1 | NC_023911 |
| pNDM102337 | JF714412 | pNDM-HN380 | JX104760 | pNDM-HF727 | NC_023914 |
| pNDM10469 | JN861072 | pNDM-KN | JN157804 | pNDM_MGR194 | NC_022740 |

**Supplementary Table 4. Result summary of recombination analysis**

| **Cluster** | **Introduction of NDM** | **Introduction of AMR genes other than NDM** | **Introduction of non-AMR genes** | **Introduction of transposable genetic elements** |
| --- | --- | --- | --- | --- |
| C1 |  |  | 1A, 1E | 1B-D, 1F |
| C2 | 2C | 2A,2B,2D | 2B-D | 2A-E |
| C3 | 3C | 3A-C | 3C | 3A, 3B, 3D-F |
| C4 |  | 4B, 4E, 4G | 4B, 4E, 4G | 4A-D, 4F-H |
| C5 |  | 5B | 5B | 5A, 5B |
| C6 | No difference found | | | |
| C7 | 7A | | | |
